# Enriched housing promotes post-stroke functional recovery through astrocytic HMGB1-IL-6-mediated angiogenesis

**DOI:** 10.1038/cddiscovery.2017.54

**Published:** 2017-08-21

**Authors:** Jia-Yi Chen, Yuan Yu, Yin Yuan, Yu-Jing Zhang, Xue-Peng Fan, Shi-Ying Yuan, Jian-Cheng Zhang, Shang-Long Yao

**Affiliations:** 1 Department of Critical Care Medicine, Union Hospital, Tongji Medical College, Huazhong University of Science and Technology, Wuhan 430022, China; 2 Institute of Anesthesia and Critical Care Medicine, Union Hospital, Tongji Medical College, Huazhong University of Science and Technology, Wuhan 430022, China; 3 Department of Critical Care Medicine, Wuhan Integrated TCM & Western Medicine Hospital, Wuhan 430022, China; 4 Department of Anesthesiology, Union Hospital, Tongji Medical College, Huazhong University of Science and Technology, Wuhan 430022, China

## Abstract

Enriched environment (EE) is shown to promote angiogenesis, neurogenesis and functional recovery after ischemic stroke. However, the underlying mechanisms remain unclear. C57BL/6 mice underwent middle cerebral artery occlusion (60 min) followed by reperfusion, after which mice were housed in either standard environment (SE) or EE. Here we found that post-ischemic EE exhibited decreased depression and anxiety-like behavior, and promoted angiogenesis and functional recovery compared to SE mice. EE mice treated with high-mobility group box-1 (HMGB1) inhibitor glycyrrhizin had an increased post-stroke depression and anxiety-like behavior, and the angiogenesis and functional recovery were decreased. HMGB1 and interleukin-6 (IL-6) expression in astrocyte were increased in EE mice. EE mice treated with glycyrrhizin decreased, whereas EE mice treated with recombinant HMGB1 (rHMGB1) increased the levels of IL-6 and p-AKT. Blockade of IL-6 with anti-IL-6-neutralizing antibody in EE mice attenuated EE-mediated angiogenesis and functional recovery. Furthermore, our *in vitro* data revealed that in primary astrocyte cultures rHMGB1 promoted the expression of IL-6 in activated astrocytes. PI_3_K/AKT signaling pathway was involved in HMGB1-mediated expression of astrocytic IL-6. Thus, our results reveal a previously uncharacterized property of HMGB1/IL-6 signaling pathway in EE-mediated angiogenesis and functional recovery after ischemic stroke.

## Introduction

Stroke is the major cause of permanent disability in adults worldwide because of the brain’s limited capacity for neural repair.^1,2^ An enriched environment (EE) has been a classic paradigm for studying the effects of a complex combination of physical, cognitive and social stimulation in rodents. EE (social interactions, voluntary and varied physical activity, and introduction of novel objects) is shown to have important roles in a normal or injured brain, ultimately beneficially influencing brain function and recovery after injury.^3–6^ EE increases neurogenesis in the adult subventricular zone (SVZ) and angiogenesis during stroke recovery,^6–8^ promotes spontaneous recovery after ischemic stroke.^7–9^ However, the underlying molecular mechanisms remain unclear.

High-mobility group box-1 protein (HMGB1) is a member of the damage-associated-molecular-pattern family of proteins that is rapidly released from necrotic neurons that amplify neuronal death in the penumbra in the acute stages of ischemic stroke.^10,11^ However, recent study suggests that astrocytic HMGB1 could promote peri-infarct angiogenesis and functional recovery in the delayed phases of stroke recovery.^12^ Previous studies have shown that EE could enhanced angiogenesis after cerebral ischemic injury.^13,14^ However, whether EE could promote angiogenesis and functional recovery through astrocytic HMGB1 during stroke recovery is unclear. One aim of our study is to investigate whether HMGB1 is an important mediator of EE on angiogenesis and long-term functional recovery after ischemic stroke.

Interleukin-6 (IL-6) belongs to the family of glycoprotein 130-activating cytokines. IL-6 has been shown to promote neuronal differentiation of neural progenitor cells (NPCs) dissociated from normal adult mice.^15^ Under physiological conditions, adult IL-6 knockout mice exhibit significantly lower NPCs survival and proliferation in the dentate gyrus and SVZ.^16^ Our previous study has found that IL-6 is essential for the promoting effects of social support on the neurogenesis and long-term outcome after ischemic stroke.^17^ A previous study demonstrates that IL-6 produced locally by resident brain cells promotes angiogenesis and affords long-term histological and functional protection after ischemic stroke.^18^ HMGB1 can bind to its receptors (glycation end products (RAGE), Toll-like receptor 2 (TLR2) and TLR4) and then trigger inflammatory cytokine expression.^19^ Evidence has shown that astrocytic HMGB1 promotes neurovascular remodeling via RAGE receptors.^12^ RAGE expression at the cell surface membrane of astrocytes mediates the expression of IL-6 in astrocytes.^20^ Thus, we speculate that astrocytic HMGB1 could promote the production and secretion of IL-6 from astrocyte in the delayed phases of stroke recovery.

In this study, we examined the hypothesis that EE could promote astrocytic HMGB1-induced production and secretion of IL-6 from astrocyte, which promoted angiogenesis and functional recovery following focal cerebral ischemia. Our study could provide a possible mechanism for explaining how EE promotes neurovascular remodeling and functional recovery after brain injury.

## Results

### EE increases the number of HMGB1-positive astrocytes in ischemic hemisphere during stroke recovery

**Figure 1:**
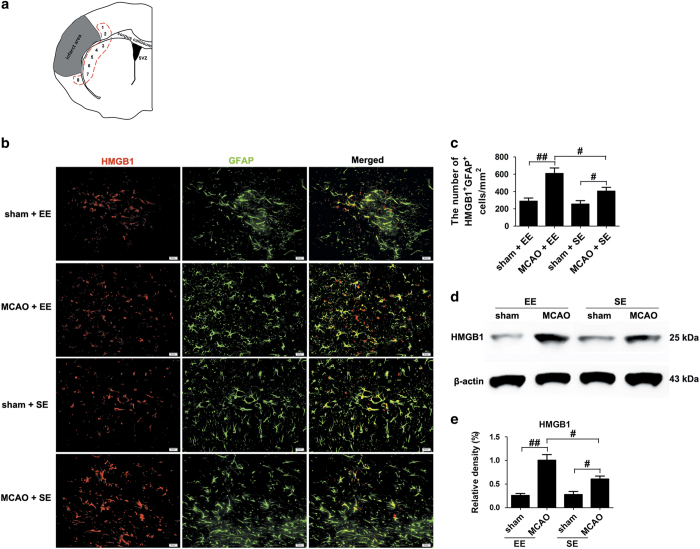
EE increases the expression of HMGB1 in astrocytes at 21 d.p.i. (**a**) Representative schematic drawing showing the sites (red dotted line) observed by IF staining. Images were acquired from each slide containing 8 fields view. (**b** and **c**) Immunofluorescence imaging and quantitative determinations of HMGB1^+^ (red) cells coexpressing GFAP (green) in the ischemic striatum at 21 d.p.i. Bar=20 *μ*m. (**d**) Western blotting for HMGB1 in the ischemic hemisphere at 21 d.p.i. (**e**) Quantitative determinations of the levels of HMGB1. Data represent mean±S.E.M., *n*=5; ^#^*P*<0.05, ^##^*P*<0.01.

As previously reported, astrocytes are the major cellular source of HMGB1 during stroke recovery.^12^ Our present study showed that the number of HMGB1 and glial fibrillary acidic protein (GFAP) double-positive cells (288±37 *versus* 609±64 and 255±40 *versus* 404±44; *P*<0.01 and *P*<0.05, respectively; Figures 1b and c) and the expression of HMGB1 (Figures 1d and e; *P*<0.01 and *P*<0.05, respectively) were all significantly increased in stroke mice regardless of housing conditions compared to their respective sham-operated group at 21 days post ischemia (d.p.i.). Post-ischemic EE significantly increased the number of HMGB1 and GFAP double-positive cells in ischemic hemisphere at 21 d.p.i., relative to standard-housed groups (404±44 *versus* 609±64, *P*<0.05; Figures 1b and c). The protein expression of HMGB1 was significantly increased in mice housing in EE at 21 d.p.i., relative to mice housed in standard environment (SE); (Figures 1d and e; *P*<0.05).

### EE mice show decreased depression and anxiety during stroke recovery

**Figure 2:**
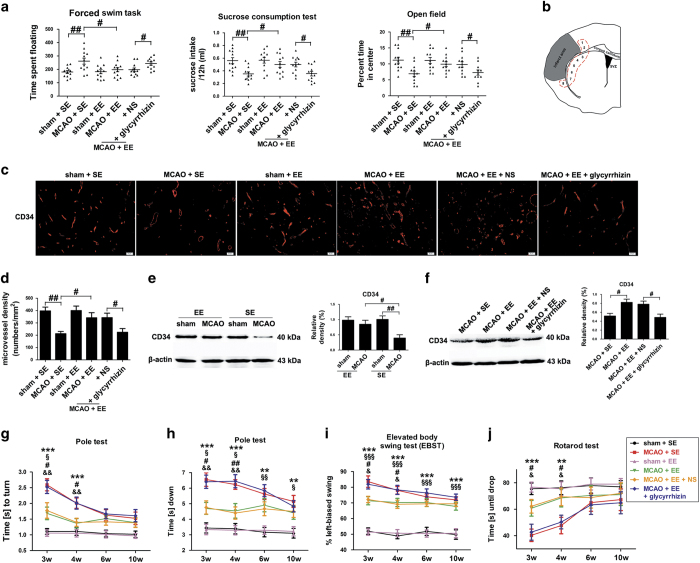
EE inhibits PSD and anxiety, and promotes angiogenesis in the peri-infarct area and functional recovery in HMGB1-dependent manner during stroke recovery. (**a**) The influences of EE on depressive and anxiety-like behavior outcome at 21 d.p.i. EE resulted in decreased time of floating in the FST, which is interpreted as decreased depressive-like behavior. EE also led to decreased levels of anxiety-like behavior, as measured by increased central tendency in the open field and the consumption of sucrose in the SCT. Data represent mean±S.E.M., *n*=12; ^#^*P*<0.05 and ^##^*P*<0.01. (**b**) Representative schematic drawing showing the sites (red dotted line) observed by IF staining. Images were acquired from each slide containing 8 fields view. (**c**) CD34 immunostaining in the peri-infarct area for each group. (**d**) The level of MVD in the brain sections of each group. Data represent mean±S.E.M., *n*=5; ^#^*P*<0.05. (**e** and **f**) Western blotting and quantitative data for CD34 in the ischemic hemisphere of each group at 21 d.p.i. Data represent mean±S.E.M., *n*=5; ^#^*P*<0.05 and ^##^*P*<0.01. Behavioral tests were assessed at 21 d.p.i., including pole test (time to turn completely head down (**g**) and time to reach the floor (**h**)), the EBST (**i**) and rotarod test (**j**). Data represent mean±S.E.M., *n*=12; **P<0.01 and ***P<0.0001, significantly different between sham-operated and MCAO-operated mice housed in SE; ^§^*P*<0.05, ^§§^*P*<0.01 and ^§§§^*P*<0.0001, significantly different between sham-operated and MCAO-operated mice housed in EE; ^#^*P*<0.05 and ^##^*P*<0.01, significantly different between enriched mice and standard housing mice; ^&^*P*<0.05 and ^&&^*P*<0.01, significantly different between glycyrrhizin-treated and NS-treated enriched mice.

Depression-like behavior was assessed with forced swim task (FST) and sucrose consumption test (SCT). There was an observed significant effect of EE on FST after ischemic stroke, as measured by spending less time floating in the FST in enriched mice, relative to mice housed in SE (Figure 2a; *P*<0.05). There was a significant effect of EE in consumption of sucrose compared to mice housing in SE (Figure 2a; *P*<0.05). However, the inhibitory effects of EE on post-stroke depression (PSD)-like behavior were attenuated by the treatment with HMGB1 inhibitor glycyrrhizin (Figure 2a; *P*<0.05).

Anxiety-like behavior was assessed with open field test (OFT). Assessment of anxiety-like behavior showed a significant effect of EE, where enriched mice spent more time exploring the center of the open field chamber, interpreted as decreased anxiety-like behavior at 21 d.p.i., relative to mice housing in SE (Figure 2a; *P*<0.05). However, the inhibitory effects of EE on post-stroke anxiety (PSA)-like behavior were attenuated by the treatment with HMGB1 inhibitor glycyrrhizin (Figure 2a; *P*<0.05).

### HMGB1 is involved in EE-mediated angiogenesis in the peri-infarct region after ischemic stroke

To study whether EE-induced inhibition of depression and anxiety-like behavior was involved in the promoting effects of HMGB1 in post-stroke angiogenesis, the microvascular density (MVD) in the peri-infarct area was assessed at 21 d.p.i. using CD34, which is expressed on early and vascular-associated tissue. Notably, EE significantly increased the CD34-positive MVD in the peri-infarct area (Figures 2c and d; 213±19 *versus* 341±42, *P*<0.05). Treatment with glycyrrhizin attenuated the promoting effects of EE on the MVD at 21 d.p.i. (Figures 2c and d; 343±36 *versus* 224±30, *P*<0.05). Western blotting analysis showed that cerebral ischemia led to a significantly decreased expression of CD34 in the peri-infarct area of SE mice (Figure 2e; *P*<0.01), but had no significantly effects on the expression of CD34 in the peri-infarct area of EE mice (Figure 2e). EE exhibited significantly higher expression of CD34 in ischemic hemisphere at 21 d.p.i. (Figure 2f; *P*<0.05). Administration of glycyrrhizin significantly decreased the expression of CD34 in enriched mice at 21 d.p.i. (Figure 2f; *P*<0.05).

We found that deficits in the pole test (Figures 2g and h), rotarod test (Figure 2i) and elevated body swing test (EBST; Figure 2j) were better in the EE 3 and 4 weeks after stroke. However, the deficits in the pole test (Figures 2g and h), rotarod test (Figure 2i) and EBST (Figure 2j) were worse in the enriched mice after treatment with glycyrrhizin.

### HMGB1 promoted the production and secretion of IL-6 from astrocytes in EE during stroke recovery

**Figure 3:**
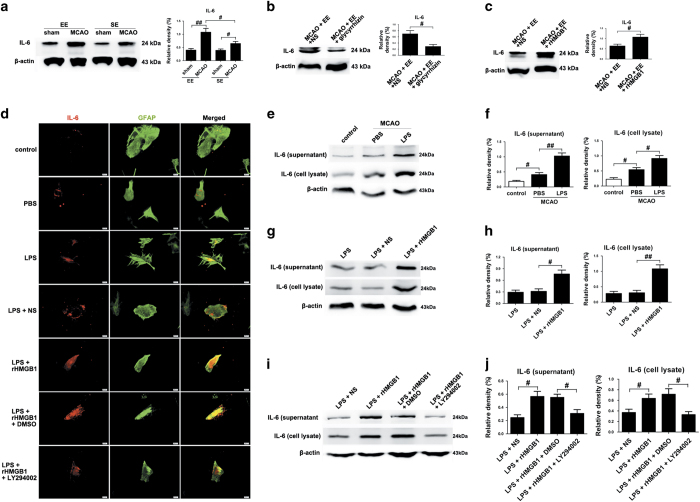
Reactive astrocytes secrete IL-6 in a HMGB1/AKT signaling-dependent manner. (**a**–**c**) Western blotting and quantitative data for IL-6 in the ischemic hemisphere for each group at 21 d.p.i. Data represent mean±S.E.M., *n*=5; ^#^*P*<0.05 and ^##^*P*<0.01. (**d**) Representative fluorescence images of primary cells stained with IL-6 (red) and GFAP (green) in cultured mouse astrocytes for each group. (**e** and **f**) Western blotting and quantitative data for IL-6 in the culture supernatant or in the cell lysates of LPS-activated mouse astrocytes. Data represent mean±S.E.M. from three experiments; ^#^*P*<0.05 and ^##^*P*<0.01. (**g** and **h**) Western blotting and quantitative data for IL-6 in the culture supernatant or in the cell lysates of LPS-activated mouse astrocytes treated with rHMGB1 or NS. Data represent mean±S.E.M. from three experiments; ^#^*P*<0.05 and ^##^*P*<0.01. (**i** and **j**) Western blotting and quantitative data for IL-6 in the culture supernatant or in the cell lysates of LPS-activated mouse astrocytes treated with rHMGB1 with or without the addition of LY294002. Data represent mean±S.E.M. from three experiments; ^#^*P*<0.05.

IL-6 expression was increased in all stroke mice regardless of housing conditions compared to their respective sham-operated group at 21 d.p.i. (Figure 3a; *P*<0.01 and *P*<0.05, respectively). However, EE exhibited significantly increased IL-6 level in ischemic hemisphere at 21 d.p.i., compared to mice housed in SE (Figure 3a; *P*<0.05). Glycyrrhizin treatment significantly decreased, whereas rHMGB1 treatment significantly increased the protein levels of IL-6 in EE mice, relative to mice housing in SE (Figures 3b and c; *P*<0.05).

Furthermore, in primary astrocytes cultures from the ischemic mice at 21 d.p.i., astrocytes were stimulated with lipopolysaccharide (LPS) to mimic a reactive phenotype. We observed a significant increase of IL-6 in both the cell lysate and culture supernatant of astrocytes treated with LPS (Figures 3d–f; *P*<0.01 and *P*<0.05, respectively). Administration of rHMGB1 further significantly increased the levels of IL-6 in both the cell lysate and culture supernatant of LPS-treated astrocytes (Figures 3d, g and h; *P*<0.01 and *P*<0.05, respectively).

### PI_3_K/AKT signaling pathway is involved in HMGB1-mediated production and secretion of IL-6 from astrocytes in EE after stroke

In primary astrocyte cultures, PI_3_K/AKT pathway inhibitor LY294002 treatment significantly attenuated the promoting effects of rHMGB1 on the expression of IL-6 in both the cell lysate and culture supernatant of LPS-treated astrocytes *in vitro* (Figures 3d, i and j; *P*<0.05).

**Figure 4:**
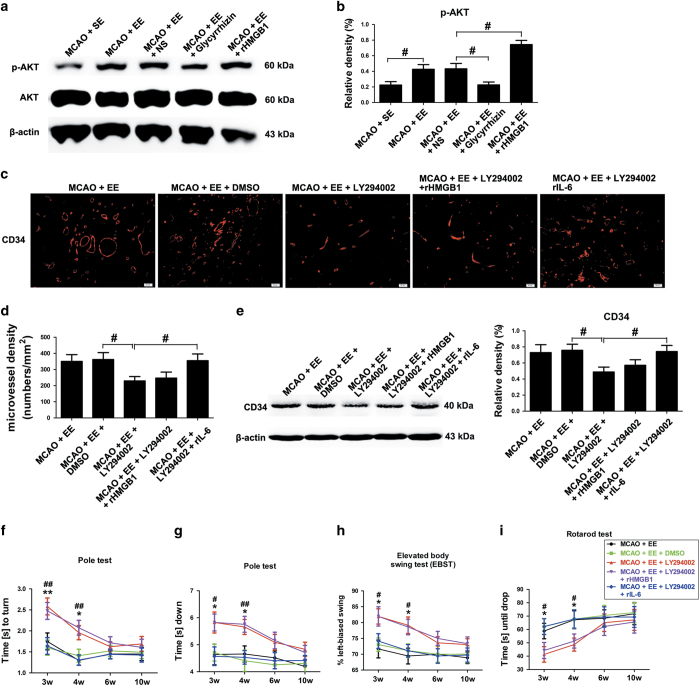
EE promotes angiogenesis in the peri-infarct area and functional recovery through AKT signaling after ischemic stroke. (**a** and **b**) Western blotting and quantitative data for p-AKT in stroke mice placed in SE or EE in the ischemic hemisphere at 21 d.p.i. Data represent mean±S.E.M., *n*=5; ^#^*P*<0.05 and ^##^*P*<0.01. (**c**) CD34 immunostaining in the peri-infarct area for each group. (**d**) The level of MVD in the brain sections of each group. Data represent mean±S.E.M., *n*=5; ^#^*P*<0.05. (**e**) Western blotting and quantitative data for CD34 in the ischemic hemisphere at 21 d.p.i. Data represent mean±S.E.M., *n*=5; ^#^*P*<0.05. Behavioral tests were assessed at 21 d.p.i, including pole test (time to turn completely head down (**f**) and time to reach the floor (**g**)), the EBST (**h**) and rotarod test (**i**). Data represent mean±S.E.M., *n*=12; *P<0.05 and **P<0.01, significantly different between LY294002-treated and DMSO-treated enriched mice; ^#^*P*<0.05 and ^##^*P*<0.01, significantly different between LY294002-treated and LY294002 plus rIL-6-treated enriched mice.

*In vivo*, we found that the expression of phospho-AKT (p-AKT) was increased in all stroke mice regardless of housing conditions compared to their respective sham-operated group at 21 d.p.i. (Figures 4a and b; *P*<0.05). Treatment with glycyrrhizin significantly decreased, whereas rHMGB1 treatment further significantly increased p-AKT levels at 21 d.p.i. in enriched mice (Figures 4a and b; *P*<0.05).

### PI_3_K/AKT signaling pathway is essential to HMGB1-mediated angiogenesis and functional recovery in enriched mice during stroke recovery

Furthermore, we found that LY294002 treatment decreased the CD34-positive MVD in the peri-infarct area at 21 d.p.i. in enriched mice even with the treatment of rHMGB1 (Figures 4c and d; 362±43 *versus* 230±27, *P*<0.05). However, enriched mice treated with rIL-6 30 min after the pre-treatment with LY294002 exhibited increased CD34-positive MVD in the peri-infarct area at 21 d.p.i. compared to enriched mice pre-treated only with LY294002 (Figures 4c and d; 230±27 *versus* 355±42, *P*<0.05). Western blotting analysis showed that EE mice treated with LY294002 exhibited significantly decreased expression of CD34 in ischemic hemisphere at 21 d.p.i. (Figure 4e; *P*<0.05). Enriched mice treated with rIL-6 30 min after the pre-treatment with LY294002 showed significantly increased CD34 protein levels at 21 d.p.i., relative to enriched mice pre-treated only with LY294002 (Figure 4e; *P*<0.05).

The deficits in the pole test (Figures 4f and g), rotarod test (Figure 4h) and EBST (Figure 4i) were worse after the administration of LY294002 in the enriched mice even with the treatment of rHMGB1 3 and 4 weeks after stroke. However, the deficits in the pole test (Figures 4f and g), rotarod test (Figure 4h) and EBST (Figure 4i) were worse in enriched mice treated with rIL-6 30 min after the pre-treatment with LY294002.

### IL-6 promoted angiogenesis and functional recovery in enriched mice after ischemic stroke

**Figure 5:**
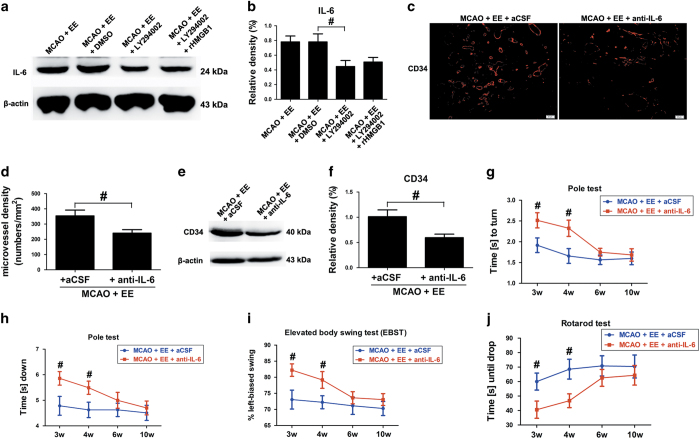
EE promotes angiogenesis in the peri-infarct area and functional recovery through HMGB1/AKT signaling-mediated IL-6 after ischemic stroke. (**a** and **b**) Western blotting and quantitative data for IL-6 in the ischemic hemisphere at 21 d.p.i. Data represent mean±S.E.M., *n*=5; ^#^*P*<0.05. (**c**) CD34 immunostaining in the peri-infarct area for each group. (**d**) The level of MVD in the brain sections of each group. Data represent mean±S.E.M., *n*=5; ^#^*P*<0.05. (**e** and **f**) Western blotting and quantitative data for CD34 in the ischemic hemisphere at 21 d.p.i. Data represent mean±S.E.M., *n*=5; ^#^*P*<0.05. Behavioral tests were assessed at 21 d.p.i., including pole test (time to turn completely head down (**g**) and time to reach the floor (**h**)), the EBST (**i**) and rotarod test (**j**). Data represent mean±S.E.M., *n*=12; ^#^*P*<0.05, significantly different between aCSF-treated and anti-IL-6-neutralizing antibody (anti-IL-6)-treated enriched mice.

Pre-treatment with LY294002 significantly decreased the expression of IL-6 in ischemic hemisphere of enriched mice even with the treatment of rHMGB1 (Figures 5a and b; *P*<0.05). Blocking IL-6 with anti-IL-6 mAbs significantly decreased the CD34-positive MVD in the peri-infarct area at 21 d.p.i. in enriched mice (Figures 5c and d; 354±38 *versus* 241±23, *P*<0.05). Western blotting analysis showed that EE mice treated with anti-IL-6 mAbs exhibited significantly reduced expression of CD34 in ischemic hemisphere at 21 d.p.i. (Figures 5e and f; *P*<0.05).

Functional assays showed that deficits in the pole test (Figures 5g and h), rotarod test (Figure 5i) and EBST (Figure 5j) were worse after treatment with anti-IL-6 in enriched mice 3 and 4 weeks after stroke.

## Discussion

**Figure 6:**
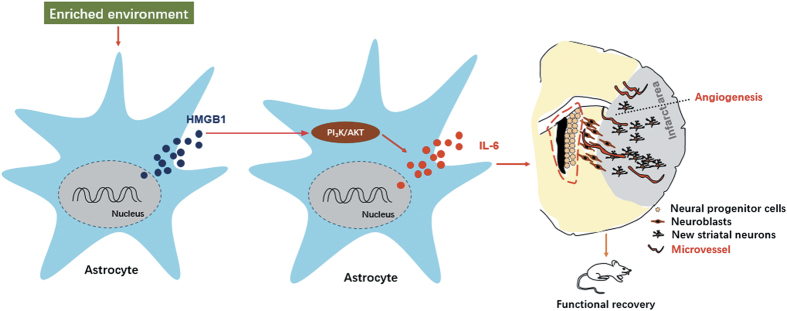
Main mechanisms of EE in post-stroke neurogenesis and functional recovery. Post-ischemic EE increased the production and secretion of activated astrocytes after ischemic stroke. Astrocytic HMGB1 then enhances the production and secretion of activated astrocytes through PI_3_K/AKT signaling pathway. Astrocytic IL-6 promotes post-stroke neurogenesis and functional recovery.

Our study confirmed that post-ischemic environmental enrichment could increase angiogenesis in the peri-infarct area and functional recovery in experimental animals during stroke recovery. PI_3_K/AKT signaling pathway was involved in HMGB1-induced IL-6 production from astrocytes in enriched mice during stroke recovery. HMGB1–AKT–IL-6 signaling pathway was involved in EE-mediated promotion of post-stroke angiogenesis and functional recovery (Figure 6).

An EE has been a classic paradigm for studying the effects of a complex combination of physical, cognitive and social stimulation in rodents, which includes running wheels, novel objects and social interactions. In many studies, an EE has exhibited therapeutic effects on stroke such as enhancement of neurogenesis in the SVZ^6^ and angiogenesis around the peri-infarction region.^7,8^ These effects have been hypothesized to lead to the promoting effects of EE on post-stroke functional recovery.^7–9^ Our current study is the first to investigate the promoting effects of EE on post-stroke angiogenesis in the peri-infarct area in mice. In our study, EE increased the CD34-positive MVD in the peri-infarct area by 60%. Evidence has shown that brain angiogenesis may provide the critical neurovascular substrates for neuronal remodeling during stroke recovery.^21^ Furthermore, one clinical study suggests that stroke patients with a higher density of blood vessels have reduced morbidity and longer survival.^22^ Therefore, EE has an important role in the neurorepair and functional recovery via promoting angiogenesis after ischemic stroke. However, the underlying molecular mechanisms are unknown. Across 51 clinical studies, approximately one-third of stroke survivors are diagnosed with PSD.^23^ About a quarter of stroke survivors are diagnosed with PSD and PSA at the same time.^24^ PSD and PSA are associated with higher morbidity and mortality, greater disability and poorer recovery after stroke.^24,25^ In this work, we found significant inhibitory effects of EE on depressive-like phenotypes in the FST and SCT, and anxiety-like behavior in the OFT after ischemic stroke. Increasing evidences have revealed that depressive- and anxiety-like behaviors have been linked to impairments of all major aspects of plasticity in the adult mammalian brain such as neurogenesis,^26,27^ gliogenesis^28,29^ and angiogenesis.^30^ Our present study found that EE significantly increased angiogenesis around the peri-infarction region. We also showed that EE significantly increased the expression of astrocytic HMGB1 in the ischemic hemisphere, which has been shown to be able to promote peri-infarct angiogenesis and functional recovery in the delayed phases of stroke recovery.^12^ Our further results showed that administration of HMGB1 inhibitor glycyrrhizin increased PSD and PSA in EE mice, and attenuated the promoting effects of EE in angiogenesis and functional recovery in the delayed phases of stroke recovery. Thus, our present data suggested that EE could promote angiogenesis and functional recovery through increasing astrocytic HMGB1 expression and subsequent inhibition of PSD and PSA during stroke recovery. However, how PSD and PSA could directly inhibit post-stroke angiogenesis and functional recovery is unclear in our present study. The intrinsic mechanisms involved in PSD- and PSA-mediated inhibition of post-stroke neurorepair should be investigated further in a future study.

A previous study has shown that IL-6 produced locally by resident brain cells could promote angiogenesis and long-term functional recovery after ischemic stroke.^18^ In our previous study, we reported that IL-6 was essential for the promoting effects of social support on the neurogenesis and long-term outcome after ischemic stroke.^17^ Evidence has shown that HMGB1 can bind to its receptors (RAGE, TLR2 and TLR4) and then trigger inflammatory cytokine expression.^19^ Astrocytic HMGB1 promotes neurovascular remodeling via RAGE receptors.^12^ RAGE expression at the cell surface membrane of astrocytes is shown to mediates the expression of IL-6 in astrocytes.^20^ On the basis of these findings, we explored the intrinsic link between astrocytic HMGB1 and IL-6 in the promoting effects of EE in angiogenesis and functional recovery after ischemic stroke. In this study, our *in vivo* results showed that astrocytic HMGB1 played an essential role in the promoting effects of EE in the expression of IL-6 in the ischemic hemisphere during stroke recovery. Our *in vitro* study confirmed that HMGB1 could promote the production and secretion of IL-6 from activated astrocytes. We further demonstrated that IL-6 promoted angiogenesis in enriched animals after ischemic stroke, indicating that the promoting effects of astrocytic HMGB1 in the post-stroke angiogenesis and functional recovery were mediated by the secretion of IL-6 from astrocytes.

Evidence has shown that IL-6 secretion by astrocytes is regulated by PI_3_K/AKT signaling in the sub-acute phases of central nervous system injury.^31^ Our and other previous studies have shown that PI_3_K/AKT signaling is the major signaling pathway implicated in NPC proliferation and differentiation in the delayed phases of stroke recovery.^32,33^ In the present study, astrocytic HMGB1-induced angiogenesis in enriched mice was dependent on PI_3_K/AKT signaling-mediated production and secretion of IL-6 from astrocytes during stroke recovery. We also found that astrocytic HMGB1-mediated inhibition of PSD and PSA could also promote angiogenesis and functional recovery. However, the intrinsic link between PSD (PSA) and AKT–IL-6 signaling in astrocytes after ischemic stroke in enriched mice is unclear and needed to be further studied.

Angiogenesis and neurogenesis holds promise for brain repair and long-term functional recovery after ischemic stroke. Angiogenesis has been shown to be coupled with neurogenesis in brain tissue repair and remodeling after ischemic stroke.^34^ Thus, pro-angiogenesis and recovery-modulating strategy are needed. In summary, our data here suggest that post-stroke EE improves stroke outcomes in an apparently pro-angiogenesis manner through astrocytic HMGB1-mediated inhibition of PSD and PSA, and also through astrocytic HMGB1-induced production and secretion of IL-6 from activated astrocytes. PI_3_K/AKT signaling is involved in astrocytic HMGB1-induced production and secretion of IL-6 from activated astrocytes in post-ischemic EE. Together, these data provide further evidence of the powerful effect that a rehabilitative strategy with EE has on the treatment of ischemic stroke.

## Materials and methods

### Animals, surgery and housing conditions

Male C57BL/6 mice (8–10 weeks old, 23–25 g) were purchased from Beijing Vital River Laboratory Animal Technology Company. Mice used for all experiments were housed under specific pathogen-free conditions at Animal Laboratory Center of Tongji Medical College. All animal experiments were performed in strict accordance with the recommendations of the Guide for the Care and Use of Laboratory Animals of the National Institutes of Health and in accordance with the ARRIVE (Animal Research: Reporting *In Vivo *Experiments) guidelines. The protocol was approved by the Animal Care and Use Committee of Tongji Medical College, Huazhong University of Science and Technology. Mice were anesthetized i.p. with ketamine (100 mg/kg) and xylazine (8 mg/kg). Focal cerebral ischemia was induced by middle cerebral artery occlusion (MCAO) with a 6-0 silicone-coated nylon monofilament for 1 h, to block the origin of the MCA as previously described.^35^ Occlusion was confirmed by laser-Doppler flowmeter (Periflux System 5000, PERIMED, Stockholm, Sweden) with a probe placed on thinned skull over the lateral parietal cortex before, during and after MCAO, as well as before death.^35^ Abrupt reduction in rCBF by ≈75–90% indicated a successful occlusion of the MCA. Mice in which ipsilateral blood flow was not reduced to <20% of the baseline after placement of the intraluminal filament and whose ipsilateral blood flow was not rapidly restored during reperfusion were excluded from subsequent experiments (≈10% in each group). Sham-operated mice were manipulated in the same way, but the MCA was not occluded. Body temperature was maintained at 37±0.5 °C with a feedback temperature control unit until the mice had recovered from surgery.

SE-housed controls were housed in a standard cage (27×22.5×14 cm^3^; 3–4 mice/cage). The EE mice were introduced in EE 2 days after MCAO or sham operation. The EE mice were housed in a spacious cage (86×76×31 cm^3^) containing novel objects such as tunnels, shelters, toys and running wheels for voluntary exercise (10 mice/cage).

### Experimental groups and drug administration

**Figure 7:**
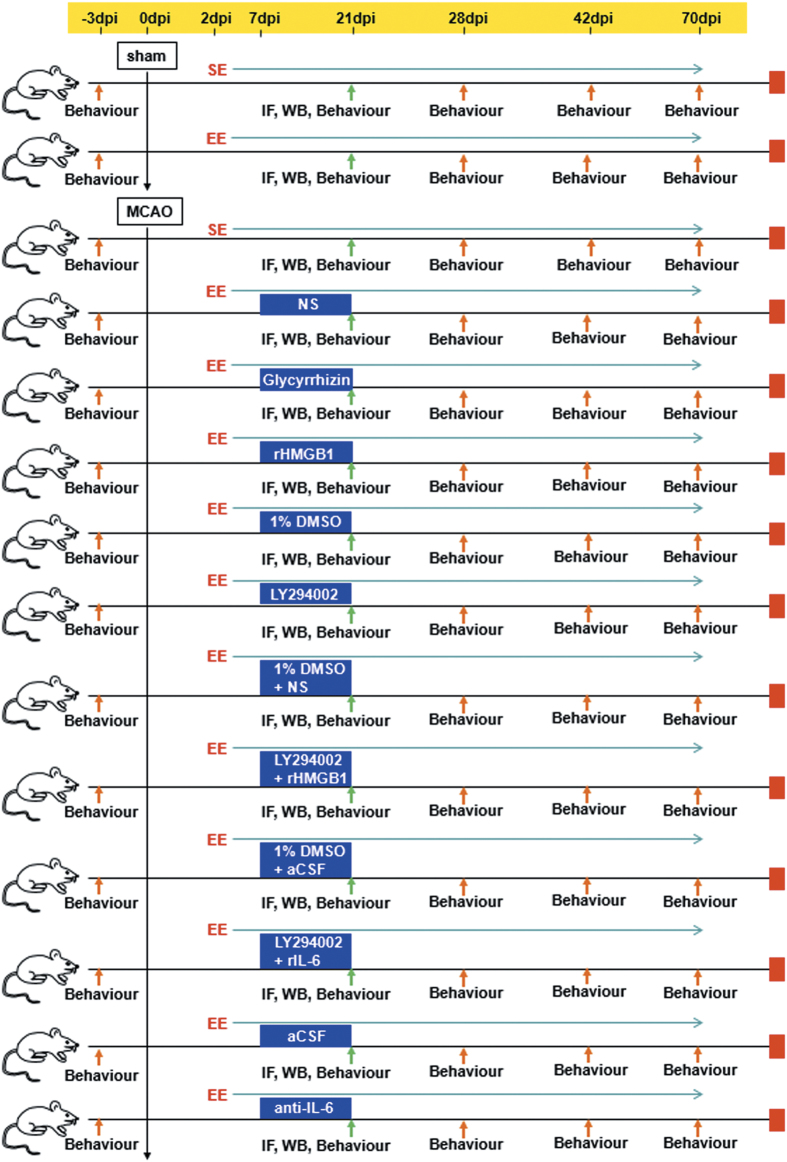
Experimental procedures and animal groups. Focal cerebral ischemia was induced by transient occlusion of the right middle cerebral artery. *n*=12/group/time point for each functional assay, and *n*=5/group for western blotting (WB) and *n*=5/group for IF. Sham, sham operation.

All treatments were administered in a blinded manner. The mice were randomly divided into nine groups (Figure 7): (1) sham operation mice housed in SE (sham+SE; *n*=12/group/time point for each functional assay, and 5/group for western blotting and 5/group for immunofluorescence (IF)); (2) sham operation mice housed in EE (sham+EE; *n*=12/group/time point for each functional assay, and 5/group for western blotting and 5/group for IF); (3) MCAO mice housed in SE (MCAO+SE; *n*=12/group/time point for each functional assay, and 5/group for western blotting and 5/group for IF); (4) MCAO mice housed in EE (MCAO+EE; *n*=12/group/time point for each functional assay, and 5/group for western blotting and 5/group for IF); (5) EE mice treated with glycyrrhizin or normal saline (NS) after MCAO (MCAO+EE+glycyrrhizin and MCAO+EE+NS; *n*=12/group/time point for each functional assay, and 5/group for western blotting and 5/group for IF): 10 mg/mouse glycyrrhizin (TCI, Shanghai, China) or NS was administrated i.p. to EE mice every 24 h for 2 weeks starting at 7 d.p.i.; (6) EE mice treated with rHMGB1 or NS after MCAO (MCAO+EE+rHMGB1 and MCAO+EE+NS; *n*=5/group for western blotting): intracerebroventricular (i.c.v.) cannulation was performed immediately after ischemia using a stereotaxic instrument (RWD Life Science Co., Ltd., Shenzhen, China), and i.c.v. injection was performed with a sterile 26-G Hamilton microsyringe (80330; Hamilton Company, Reno, NV, USA). EE mice were injected i.c.v. with 2 *μ*g rHMGB1 (R&D Systems, Minneapolis, MN, USA) diluted in NS every 24 h for 2 weeks starting at 7 d.p.i.; (7) EE mice treated with PI_3_K/AKT pathway inhibitor LY294002 or 1% DMSO after MCAO (MCAO+EE+LY294002 and MCAO+EE+DMSO; *n*=12/group/time point for each functional assay, and 5/group for western blotting and 5/group for IF): LY294002 (200 nM in 0.5 *μ*l of 1% DMSO; Sigma-Aldrich, St. Louis, MO, USA) or equal amount of 1% DMSO was i.c.v. injected to EE mice daily for 14 days, starting at 7 d.p.i.; (8) EE mice treated with LY294002 plus rHMGB1 or rIL-6 after MCAO (MCAO+EE+LY294002+rHMGB1 and MCAO+EE+LY294002+rIL-6; *n*=12/group/time point for each functional assay, and 5/group for western blotting and 5/group for IF): 2 *μ*l drops of rIL-6 (R&D Systems) diluted in NS (0.01 *μ*g/*μ*l) was intranasally administrated to alternating nostrils of restrained conscious mice with a 2 min interval between applications. Drops were placed at the opening of the nostril, allowing the mice to snort each drop into the nasal cavity. A total of 10 *μ*l of dose solution, containing 0.1 *μ*g rIL-6 was delivered over a course of 5 min. The injection of rIL-6 (or vehicle) was repeated every 24 h for 2 weeks starting at 7 d.p.i. LY294002 was i.c.v. injected to EE mice 30 min before rHMGB1 or rIL-6 treatment daily for 14 days, starting at 7 d.p.i.; (9) EE mice treated with anti-IL-6-neutralizing antibodies (anti-IL-6 mAbs) or artificial cerebrospinal fluid (aCSF) after MCAO (MCAO+EE+anti-IL-6 and MCAO+EE+aCSF; *n*=12/group/time point for each functional assay, and 5/group for western blotting and 5/group for IF): EE mice received i.c.v. either anti-IL-6 mAbs (10 ng in 2 *μ*l aCSF; R&D Systems) or 2 *μ*l aCSF. This dose has been used successfully to neutralize IL-6 signaling in mice.^36^

### Functional assays

EBST was performed to evaluate the symmetry of motor behavior 3, 4, 6 and 10 weeks after stroke.^37^ Mice (*n*=12/group/time point) were examined for lateral movements/turning when their bodies were suspended 10 cm above the testing table by lifting their tails. A swing was recorded when mice moved their head away from the vertical axis (angle >10°) in three sets of 10 trials, performed over 5 min. Results are expressed as the ratio of total number of contralateral swings.

The rotarod test provided an index of forelimb and hindlimb motor coordination and balance.^37^ Mice (*n*=12/group/time point) were trained daily on an accelerating (5–40 r.p.m.) rotating rod for 3 days before MCAO; only those mice able to remain on the rod for 20 s at 40 r.p.m. were subjected to MCAO. Test sessions consisting of three trials at 40 r.p.m. were carried out 3, 4, 6 and 10 weeks after stroke, by an investigator who was blinded to the experimental groups. The final score was expressed as the mean time that a mouse could remain on the rod over three trials.

The pole test was used to assess forelimb strength, ability to grasp and balance performed in a blinded fashion 3, 4, 6 and 10 weeks after stroke.^18^ Mice (*n*=12/group/time point) were placed head upward near the top of a vertical steel pole (60 cm high with rough surface). Thereafter, both time taken to orientate the body completely downwards and to reach the floor with all four paws were recorded.

*FST*: To assess depression-like behavior at 3 weeks after stroke, mice (*n*=12/group) were placed into an opaque cylinder tank (24 cm in diameter and 53 cm high) filled to a depth of 30 cm with water (25±1 °C). Swimming behavior was recorded for 5 min and scored for time spent actively swimming *versus* floating. Quantification of float *versus* swim time was performed with Observer software (Version 5; Exeter Software, New York, NY, USA).

SCT was used to assess depression-like behavior at 3 weeks after stroke. Mice (*n*=12/group) are presented with the option of consuming either water or a 3% sucrose solution. Two identical 10 ml vials were placed on a custom-made wire cage top. At 3 days before MCAO, each individual mouse was provided two 10 ml vials of water for 12 h. The following morning, all mice were returned to their original housing condition and provided their normal drinking water and cage tops. That evening mice were again provided with two 10 ml vials of 3% sucrose overnight. After completion of habituation, all mice were again returned to their original housing condition and provided their normal drinking water and cage tops. On the day of testing, mice were water-deprived in their original housing condition for 6 h, then kept in a new cage with the customized cage top and one 10 ml vial of water and one 10 ml vial of 3% sucrose solution overnight. The volume of consumption of both solutions was recorded the following day by an observer blinded to housing condition.

*OFT*: Anxiety-like behavior of mice (*n*=12/group) were assessed during a 60 min session in an open field apparatus (40×40×37.5 cm) using Flex Field photobeam activity (San Diego, CA, USA) at 3 weeks after stroke. Data were analyzed to determine the relative amount of activity occurring in the periphery *versus* the center of the apparatus (anxiety-like behavior).

### *In vitro* LPS stimulation and treatments in the astrocyte culture

Cells were dissociated from ischemic hemispheric brains at 21 d.p.i. or from sham-operated brains as described in our previous study.^38^ Purified astrocytes were treated with LPS (100 ng/ml; Sigma, St. Louis, MO, USA) in the presence or absence of pre-treatment with LY294002 (200 nM, 30 min before LPS treatment). rHMGB1 (500 ng/ml) was added to the media 30 min after LY294002 treatment. At 16 h after treatment, the cells were fixed with 4% paraformaldehyde and subjected to histological analysis. The supernatant of cell culture was collected for western blotting analysis.

### Western blotting

Western blotting was done according to our previously established protocols.^11,35^ The primary cells were lysed in radioimmunoprecipitation assay buffer containing protease and phosphatase inhibitors (KeyGen Biotech, Nanjing, China). Cell culture supernatant proteins were extracted as previously described.^39^ Equal amounts of protein were subjected to SDS-PAGE analysis, transferred onto PVDF membrane and probed with primary antibodies against rabbit polyclonal HMGB1 (1 : 1000, Abclonal Technology, Wuhan, China), rabbit polyclonal CD34 (1 : 1000, Abclonal), rabbit polyclonal IL-6 (1 : 1000, Abclonal), rabbit polyclonal p-AKT (1 : 1000, Cell Signaling Technology, Danvers, MA, USA), rabbit polyclonal total-AKT (1 : 1000, Cell Signaling Technology) or mouse monoclonal *β*-actin (1 : 2000; Santa Cruz Biotechnology, Santa Cruz, CA, USA). After washing, the membranes were treated with horseradish peroxidase-conjugated goat anti-mouse or goat anti-rabbit IgG antibody (1 : 3000; Proteintech Group, Inc., Wuhan, China). Chemiluminescence detection was carried out with electrochemiluminescence Western Blotting Detection Reagents (Millipore, Billerica, MA, USA) plus BioWest enhanced chemiluminescence (UVP, Upland, CA, USA). Band intensity was quantified with ImageJ software (NIH, Bethesda, MD, USA).

### Immunofluorescence

Mice were transcardially perfused with 4% paraformaldehyde, and the brains were paraffin-embedded. Brains were cut into 4 *μ*m-thick coronal sections. Cell cultures were fixed using 4% paraformaldehyde for 15 min. For both sections and cells, nonspecific binding was blocked using normal goat serum. Immunoassays were performed using the following antibodies: rabbit polyclonal HMGB1 (1 : 20, Abclonal); mouse monoclonal GFAP (1 : 300, Cell Signaling Technology); rabbit polyclonal IL-6 (1 : 20, Abclonal); or rabbit polyclonal CD34 (1 : 20, Abclonal). Primary antibodies were detected with dylight 549-conjugated goat anti-rabbit, dylight 488-conjugated goat anti-mouse secondary antibody. For the quantitative analysis, eight non-overlapping fields in the peri-infarct area per slide at a magnification of ×400 were recorded by an observer who was blinded to the experimental groups. The mean volume of MVD from five sequential brain sections of individual mouse was calculated and expressed as numbers/mm^2^.

### Data analysis

Multiple comparisons were performed by one-way ANOVA followed by Newman–Keuls multiple comparison tests for multiple comparisons (GraphPad Prism statistics software version 5.0, La Jolla, CA, USA). Two groups were compared by two-tailed Student’s *t*-test. Behavioral data were analyzed by two-way ANOVA with repeated measures, followed by *post hoc* multiple comparison tests. All data are presented as mean±S.E.M. The *P*-values <0.05 were considered statistically significant.

## Publisher’s note

Springer Nature remains neutral with regard to jurisdictional claims in published maps and institutional affiliations.
